# Seroprevalence of SARS-CoV-2 antibodies among healthcare workers in Dutch hospitals after the 2020 first wave: a multicentre cross-sectional study with prospective follow-up

**DOI:** 10.1186/s13756-023-01324-x

**Published:** 2023-11-29

**Authors:** Claudia Recanatini, Corine H. GeurtsvanKessel, Suzan D. Pas, Els M. Broens, Martje Maas, Rosa van Mansfeld, Anne J. G. Mutsaers-van Oudheusden, Miranda van Rijen, Emile F. Schippers, Arjan Stegeman, Adriana Tami, Karin Ellen Veldkamp, Hannah Visser, Andreas Voss, Marjolijn C. A. Wegdam-Blans, Heiman F. L. Wertheim, Peter C. Wever, Marion P. G. Koopmans, Jan A. J. W. Kluytmans, Marjolein F. Q. Kluytmans-van den Bergh, COCON Study Group, Anneke Bergmans, Wouter van den Bijllaardt, Els Broens, Anton Buiting, Wietske Dohmen, Alexander Friedrich, Corine GeurtsvanKessel, Bernardina van der Gun, Dick Heederik, Menno de Jong, Jan Kluytmans, Marjolein Kluytmans-van den Bergh, Marion Koopmans, Martje Maas, Rosa van Mansfeld, Angelique Meijer, Jean-Luc Murk, Marrigje Nabuurs, Bert Niesters, Jaap ten Oever, Anne Mutsaers-van Oudheusden, Suzan Pas, Claudia Recanatini, Miranda van Rijen, Emile Schippers, Valentijn Schweitzer, Arjan Stegeman, Roel Streefkerk, Adriana Tami, Karin Ellen Veldkamp, Alida Veloo, Hannah Visser, Andreas Voss, Jutte de Vries, Marjolijn Wegdam-Blans, Heiman Wertheim, Peter Wever, Karin Wold, Herman Wunderink

**Affiliations:** 1 Julius Center for Health Sciences and Primary Care, University Medical Center Utrecht, Utrecht University, Utrecht, The Netherlands; 2 Viroscience Department, Erasmus University Medical Center, Rotterdam, The Netherlands; 3 Microvida Laboratory for Medical Microbiology, Bravis Hospital, Roosendaal, The Netherlands; 4 Department Biomolecular Health Sciences, Faculty of Veterinary Medicine, Utrecht University, Utrecht, The Netherlands; 5 Department of Internal Medicine, Bernhoven Hospital, Uden, The Netherlands; 6 Department of Medical Microbiology, Amsterdam University Medical Centers, University of Amsterdam, Amsterdam, The Netherlands; 7 Department of Infection Control, Elisabeth-TweeSteden Hospital, Tilburg, The Netherlands; 8 Department of Infection Control, Amphia Hospital, Breda, The Netherlands; 9 Department of Internal Medicine, Haga Hospital, The Hague, The Netherlands; 10 Department of Population Health Sciences, Faculty of Veterinary Medicine, Utrecht University, Utrecht, The Netherlands; 11 Department of Medical Microbiology and Infection Prevention, University of Groningen, University Medical Center Groningen, Groningen, The Netherlands; 12 Department of Medical Microbiology, Leiden University Medical Center, Leiden, The Netherlands; 13 Department of Internal Medicine, Beatrix Hospital, Gorinchem, The Netherlands; 14 Department of Medical Microbiology and Infectious Diseases, Canisius Wilhelmina Hospital, Nijmegen, The Netherlands; 15 Radboud Center for Infectious Diseases, Radboud University Medical Center, Nijmegen, The Netherlands; 16 Present Address: Department of Medical Microbiology, University Medical Center Utrecht, Utrecht University, Utrecht, The Netherlands; 17 Catharina Hospital, Eindhoven, The Netherlands; 18 Hospital St. Jans Gasthuis, Weert, The Netherlands; 19 Department of Medical Microbiology, Stichting PAMM, Veldhoven, The Netherlands; 20 Department of Medical Microbiology and Infection Control, Jeroen Bosch Hospital, ’s Hertogenbosch, The Netherlands; 21 Amphia Academy Infectious Disease Foundation, Amphia Hospital, Breda, The Netherlands; 22 Division of Environmental Epidemiology, Institute for Risk Assessment Sciences, Utrecht University, Utrecht, The Netherlands; 23 Jeroen Bosch Hospital, ’s Hertogenbosch, The Netherlands; 24 Microvida, Elisabeth-TweeSteden Hospital, Tilburg, The Netherlands; 25 Department of Medical Microbiology, Division of Clinical Virology, University Medical Center Groningen, Groningen, The Netherlands; 26 Regional Laboratory for Medical Microbiology, Dordrecht, The Netherlands; 27 Present Address: Department of Medical Microbiology, Radboud University Medical Center, Nijmegen, The Netherlands

**Keywords:** Seroprevalence, SARS-CoV-2, COVID-19, Antibodies, Risk factor, Self-reported symptoms, Healthcare worker

## Abstract

**Background:**

We aimed to estimate the severe acute respiratory syndrome coronavirus 2 (SARS-CoV-2) seroprevalence and describe its determinants and associated symptoms among unvaccinated healthcare workers (HCWs) after the first wave of the pandemic.

**Methods:**

HCWs from 13 Dutch hospitals were screened for antibodies against the spike protein of SARS-CoV-2 in June-July 2020 and after three months. Participants completed a retrospective questionnaire on determinants for occupational and community exposure to SARS-CoV-2 and symptoms suggestive of COVID-19 experienced since January 2020. The seroprevalence was calculated per baseline characteristic and symptom at baseline and after follow-up. Adjusted odds ratios (aOR) for seropositivity were determined using logistic regression.

**Results:**

Among 2328 HCWs, 323 (13.9%) were seropositive at enrolment, 49 of whom (15%) reported no previous symptoms suggestive of COVID-19. During follow-up, only 1% of the tested participants seroconverted. Seroprevalence was higher in younger HCWs compared to the mid-age category (aOR 1.53, 95% CI 1.07–2.18). Nurses (aOR 2.21, 95% CI 1.34–3.64) and administrative staff (aOR 1.87, 95% CI 1.02–3.43) had a higher seroprevalence than physicians. The highest seroprevalence was observed in HCWs in the emergency department (ED) (aOR 1.79, 95% CI 1.10–2.91), the lowest in HCWs in the intensive, high, or medium care units (aOR 0.47, 95% CI 0.31–0.71). Chronic respiratory disease, smoking, and having a dog were independently associated with a lower seroprevalence, while HCWs with diabetes mellitus had a higher seroprevalence. In a multivariable model containing all self-reported symptoms since January 2020, altered smell and taste, fever, general malaise/fatigue, and muscle aches were positively associated with developing antibodies, while sore throat and chills were negatively associated.

**Conclusions:**

The SARS-CoV-2 seroprevalence in unvaccinated HCWs of 13 Dutch hospitals was 14% in June-July 2020 and remained stable after three months. A higher seroprevalence was observed in the ED and among nurses, administrative and young staff, and those with diabetes mellitus, while a lower seroprevalence was found in HCWs in intensive, high, or medium care, and those with self-reported lung disease, smokers, and dog owners. A history of altered smell or taste, fever, muscle aches and fatigue were independently associated with the presence of SARS-CoV-2 antibodies in unvaccinated HCWs.

**Supplementary Information:**

The online version contains supplementary material available at 10.1186/s13756-023-01324-x.

## Background

In 2020, hospitals worldwide were overburdened with patients with coronavirus disease 2019 (COVID-19), and healthcare workers (HCWs) were at high risk of acquiring an infection with the new severe acute respiratory syndrome coronavirus 2 (SARS-CoV-2) [1–4]. HCWs were considered vulnerable, especially during the early phase of the pandemic [5], before transmission dynamics were fully recognised and when the availability of personal protective equipment (PPE) was limited [6, 7]. In April 2020, the median percentage of HCW infections among total COVID-19 cases was reported to be 10% (range 1–24%) across 40 countries [8]. In addition, a study from the United Kingdom and the United States of America reported that frontline HCWs had a 3.4 times higher risk for SARS-CoV-2 infection than people in the community during the first wave [9].

Seroepidemiology studies can help uncover the burden of disease, including the rate of asymptomatic infections, and provide better estimates of the incidence of disease [10]. According to two systematic reviews with meta-analysis [11, 12], the SARS-CoV-2 seroprevalence among HCWs in 2020, before vaccinations started, was 8.0% and 8.7%, respectively, with differences between countries. In the Netherlands, the seroprevalence during and after the first epidemic wave was estimated to be 2.8% (March 2020) and 4.5% (June 2020) in the community, and 3.4% (April 2020) and 5.9% (May 2020) in healthy plasma donors [13–16]. Two single-centre seroprevalence studies were performed in Dutch HCWs, reporting the presence of antibodies in 21.1% of the staff of a teaching hospital in a high endemic region in June 2020 [17] and 9.0% of the staff of two tertiary care hospitals, respectively [18].

The risk of SARS-CoV-2 acquisition attributed to exposure in the healthcare setting, including whether the risk differs between staff functions, has been studied with conflicting results [11, 19, 20]. Some studies suggested that frontline HCWs or those caring for SARS-CoV-2-positive patients are at increased risk [3, 18, 19], while others highlighted the substantial contribution of community exposure to the overall transmission risk in HCWs [19, 21–23]. Furthermore, data are lacking for the Dutch hospital setting.

The objective of this multicentre study was to estimate the SARS-CoV-2 seroprevalence among unvaccinated HCWs in Dutch hospitals after the first wave of the COVID-19 pandemic in 2020 and describe the cumulative incidence of seroconversion and seroreversion after three months of follow-up. Additionally, we sought to identify determinants associated with seropositivity on which to base hospital infection control policies. Finally, we aimed to estimate the rate of asymptomatic infections and describe the occurrence of self-reported symptoms associated with developing SARS-CoV-2 antibodies.

## Methods

### Study design and data collection

The COCON (Control of COVID-19 in hospitals) study was a cross-sectional study with prospective follow-up enrolling HCWs of 13 Dutch university and non-university hospitals, with representative participation of hospitals from areas with different COVID-19 incidences. Healthcare workers aged 18 years or above were recruited from the population of hospital employees and enrolled between June 3 and July 10, 2020. Participation was voluntary, and each site could enrol 200 participants.

After providing written informed consent, participants filled in a retrospective questionnaire on demographics and factors potentially related to professional exposure (hospital staff role, working department, direct patient contact) and community exposure (history of travel, household size, living with children, owning a pet) to SARS-CoV-2 since January 1, 2020. Also, chronic conditions (diabetes mellitus, chronic respiratory disease, cardiovascular disease, immune disorder), medication use (non-steroidal anti-inflammatory drugs, immunosuppressant medication, antihypertensive medication), influenza vaccination for the season 2019–2020, previous Bacillus Calmette–Guérin (BCG) vaccination and smoking habits were investigated. Results of any SARS-CoV-2 polymerase chain reaction (PCR) tests performed since January 1, 2020, and reasons for having been tested were collected. HCWs were also asked to report any symptoms suggestive of COVID-19 experienced since January 1, 2020, including the date of onset and duration of symptoms and whether they were hospitalised. Participants who had not been tested were asked whether they believed they had had COVID-19. The list of symptoms suggestive of COVID-19 included: fever (≥ 38.0 °C), chills, coughing, shortness of breath, severe myalgia, general malaise, sore throat, runny nose, painful eyes, headache, chest pain (retrosternal or subscapular), abdominal pain, diarrhoea, and altered or decreased smell or taste.

At enrolment and three months after enrolment, a blood sample was drawn to determine the HCWs’ SARS-CoV-2 serostatus at the end of the first wave and during the summer of 2020, respectively.

### Assessment of infection and serological status

Serological analyses for SARS-CoV-2 antibodies were performed at the Department of Viroscience, Erasmus MC, Rotterdam, the Netherlands. Serum samples were tested for the presence and levels of total Ig antibodies against the SARS-CoV-2 spike protein by enzyme-linked immunosorbent assay (ELISA, Wantai Biological Pharmacy Enterprise Co., Beijing, China) [24]. As indicated by the manufacturer, samples with an optical density (OD) ratio above 1.0 were interpreted as positive. At enrolment, positive ELISA results were followed by confirmatory testing for their neutralisation capacity against SARS-CoV-2 using the 50% plaque reduction neutralisation test (PRNT50) [25]. The PRNT50 titre was defined as the reciprocal value of the highest serum dilution resulting in 50% plaque reduction. Based on assay validation, serum samples with a PRNT50 titre equal to or greater than 20 were considered SARS-CoV-2 positive [25].

### Statistical analysis

The presence of SARS-CoV-2 neutralising antibodies at baseline was considered the primary outcome. Univariable logistic regression was used to examine the association between SARS-CoV-2 seropositivity and each determinant. A mixed-effects logistic regression model, with a random intercept to account for clustering by hospital, was then fitted to calculate adjusted odds ratios (aOR) and quantify the associations between serostatus and demographic, personal and occupational factors. The selection of variables included in the model was based on clinical reasoning and their theoretical role as a possible risk factor or confounder while trying to minimise collinearity between variables. No model building or comparison was performed, as our purpose was to evaluate the role of specific risk factors and not generate a prediction model. Demographics and variables related to professional and community exposure were included in the analysis. The differences in SARS-CoV-2 incidence across the country were taken into account by adding the variable province as a fixed effect to the multivariable model.

Similarly, univariable and multivariable logistic regression analyses were performed to assess independent associations between each self-reported symptom and the development of neutralising antibodies.

Continuous variables are reported as mean and standard deviation or median and interquartile range (IQR), as appropriate. Categorical variables are reported as count and percentage.

Fifteen percent of the HCWs declared that their BCG vaccine status was unknown; therefore, the variable was excluded from the multivariable analysis. There were no missing values in the other variables. Thus, a complete case analysis was performed. A sensitivity analysis was performed using seropositivity based on total SARS-CoV-2 antibodies as the outcome variable. Statistical analyses were performed with R version 4.1.2 (2021-11-01) and SPSS statistical software version 26.

### Sample size calculation

The number of deaths due to COVID-19 in the population, as reported by the National Institute for Public Health and the Environment (RIVM) on April 7, 2020, was 2,101. The actual number of deaths was estimated to be at least two times higher than the number reported since not all COVID-19 patients outside the hospitals were tested [26]. Therefore, the case fatality rate was estimated to be between 0.5 and 1.0%. Based on these estimates, it was expected that 400,000 to 800,000 Dutch inhabitants had been affected at baseline. The seroprevalence was, therefore, expected to be between 2.5 and 5.0%. Taking the highest estimate for seroprevalence (5.0%) and a 2% width for the two-sided 95% confidence interval resulted in a required sample size of 1825 [27]. After accounting for an expected 10% loss of blood samples for the primary endpoint analysis, the total number of subjects to be enrolled was set at 2000.

## Results

### Baseline characteristics

**Fig. 1:**
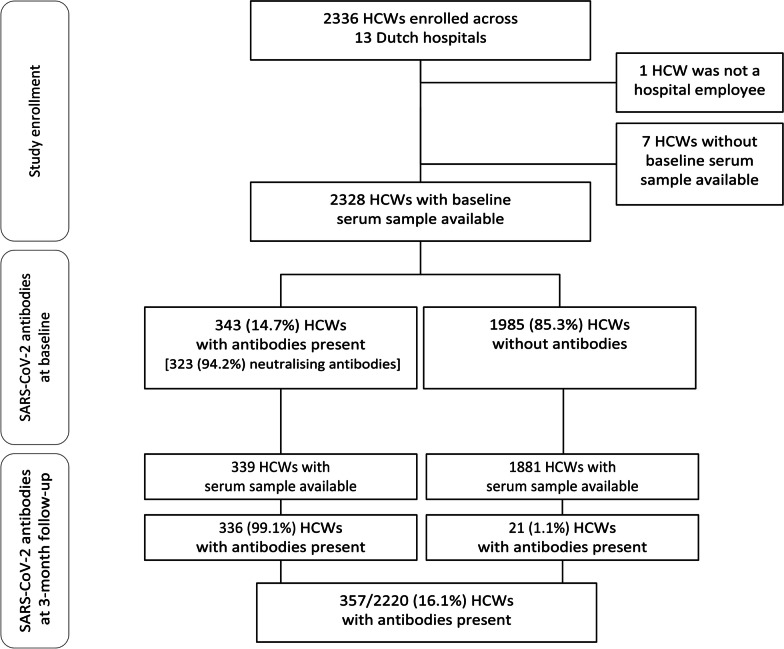
Flowchart of enrolled participants and serological findings. HCWs: healthcare workers

A total number of 2335 HCWs was enrolled in 13 participating Dutch hospitals located in six Dutch provinces. Of those, 2328 (99.7%) had a blood sample drawn to assess the presence of anti-SARS-CoV-2 antibodies at the time of study enrolment and were included in the analysis (Fig. 1). The median age of included subjects was 43 years (IQR: 33–53), and 1898 (81.5%) were women (Table 1). The study population comprised 282 physicians (12.1%), 731 nurses (31.4%), 499 administrative staff (21.4%), and 816 (35.1%) other supporting staff. More than half (n = 1212, 52.1%) of the HCWs reported having had protected or unprotected direct contact (< 1.5 m) with a COVID-19 patient. One hundred sixty-six (7.1%) reported suffering from chronic respiratory disease (mainly asthma, n = 146, 88.0%), and 184 (7.9%) from chronic cardiovascular disease (mainly hypertension, n = 106, 57.6%), 1064 (45.7%) were immunised against influenza in the previous season (2019/2020). Regarding the household composition, 266 (11.4%) HCWs were living alone, 787 (33.8%) reported living together with one person, and 1275 (54.8%) were sharing the household with more people; 668 (28.7%) participants reported having in the household at least one child aged 11 years or younger. Four hundred fifty (19.3%) had at least one dog, and 522 (22.4%) had at least one cat.

**Table 1:** Distribution of population characteristics by serostatus at baseline (n and %)

Characteristic		Total (n = 2328)	SARS-CoV-2 serology Neutralising antibodies at baseline	
			Seronegative (n = 2005)	Seropositive (n = 323)
Gender	Female	1898 (81.5)	1629 (81.2)	269 (83.3)
	Male	430 (18.5)	376 (18.8)	54 (16.7)
Age category	≤ 35	757 (32.5)	642 (32.0)	115 (35.6)
	36–49	756 (32.5)	673 (33.6)	83 (25.7)
	≥ 50	815 (35.0)	690 (34.4)	125 (38.7)
Hospital staff role	Physician	282 (12.1)	254 (12.7)	28 (8.7)
	Nurse	731 (31.4)	600 (29.9)	131 (40.6)
	Administrative	499 (21.4)	428 (21.3)	71 (22.0)
	Other	816 (35.1)	723 (36.1)	93 (28.8)
Direct contact with patients*	No patient contact	612 (26.3)	540 (26.9)	72 (22.3)
	With non-COVID patients	504 (21.6)	441 (22.0)	63 (19.5)
	With COVID patient	1212 (52.1)	1024 (51.1)	188 (58.2)
COVID-19 dedicated ward	Intensive to medium care	347 (14.9)	314 (15.7)	33 (10.2)
	Emergency	145 (6.2)	116 (5.8)	29 (9.0)
	Infectious disease	149 (6.4)	125 (6.2)	24 (7.4)
	Pulmonology	203 (8.7)	174 (8.7)	29 (9.0)
Smoker	No	1492 (64.1)	1269 (63.3)	223 (69.0)
	Former	653 (28.0)	567 (28.3)	86 (26.6)
	Current	183 (7.9)	169 (8.4)	14 (4.3)
Chronic respiratory disease	No	2162 (92.9)	1851 (92.3)	311 (96.3)
	Yes	166 (7.1)	154 (7.7)	12 (3.7)
Diabetes Mellitus	No	2296 (98.6)	1980 (98.8)	316 (97.8)
	Yes	32 (1.4)	25 (1.2)	7 (2.2)
Cardiovascular disease	No	2144 (92.1)	1852 (92.4)	292 (90.4)
	Yes	184 (7.9)	153 (7.6)	31 (9.6)
Immune disorder	No	2277 (97.8)	1961 (97.8)	316 (97.8)
	Yes	51 (2.2)	44 (2.2)	7 (2.2)
Use of NSAID	No	2264 (97.3)	1946 (97.1)	318 (98.5)
	Yes	64 (2.7)	59 (2.9)	5 (1.5)
Use of antihypertensive medications	No	2183 (93.8)	1880 (93.8)	303 (93.8)
	Yes	145 (6.2)	125 (6.2)	20 (6.2)
Use of immunosuppressants	No	2271 (97.6)	1951 (97.3)	320 (99.1)
	Yes	57 (2.4)	54 (2.7)	3 (0.9)
BCG vaccine received between 1940 and 2020	No	1739 (74.7)	1505 (75.1)	234 (72.4)
	Yes	251 (10.8)	221 (11.0)	30 (9.3)
	Unknown	338 (14.5)	279 (13.9)	59 (18.3)
Influenza vaccine season 2019/20	No	1264 (54.3)	1063 (53.0)	201 (62.2)
	Yes	1064 (45.7)	942 (47.0)	122 (37.8)
Travel to other European country*	No	1699 (73.0)	1472 (73.4)	227 (70.3)
	Yes	629 (27.0)	533 (26.6)	96 (29.7)
Number of household members^#^	1	266 (11.4)	227 (11.3)	39 (12.1)
	2	787 (33.8)	688 (34.3)	99 (30.7)
	3	352 (15.1)	307 (15.3)	45 (13.9)
	4	630 (27.1)	535 (26.7)	95 (29.4)
	≥ 5	293 (12.6)	248 (12.4)	45 (13.9)
Children ≤ 11 yrs in the household	No	1660 (71.3)	1419 (70.8)	241 (74.6)
	Yes	668 (28.7)	586 (29.2)	82 (25.4)
Dog owner	No	1878 (80.7)	1607 (80.1)	271 (83.9)
	Yes	450 (19.3)	398 (19.9)	52 (16.1)
Cat owner	No	1806 (77.6)	1541 (76.9)	265 (82.0)
	Cat outside	393 (16.9)	348 (17.4)	45 (13.9)
	Cat inside	129 (5.5)	116 (5.8)	13 (4.0)
Province of residence	North Brabant	927 (39.8)	746 (37.2)	181 (56.0)
	North Holland	137 (5.9)	121 (6.0)	16 (5.0)
	South Holland	554 (23.8)	497 (24.8)	57 (17.6)
	Gelderland	353 (15.2)	323 (16.1)	30 (9.3)
	Limburg	200 (8.6)	166 (8.3)	34 (10.5)
	Groningen	157 (6.7)	152 (7.6)	5 (1.5)
SARS-CoV-2 RT-PCR	Symptomatic			
	Positive	112 (16.8)	16 (3.1)	96 (65.8)
	Negative	556 (83.2)	506 (96.6)	50 (34.2)
	Asymptomatic			
	Positive	0 (0.0)	0 (0.0)	0 (0.0)
	Negative	52 (100.0)	46 (100.0)	6 (100.0)

*BCG* Bacillus Calmette-Guérin, *NSAID* non-steroidal anti-inflammatory drugs, *RT-PCR* Reverse transcription-polymerase chain reaction

*Since January 1, 2020. ^#^Including study participant

### SARS-CoV-2 seroprevalence at baseline and 3-month follow-up

At baseline, SARS-CoV-2 total and neutralising antibodies were detected in 343/2328 (14.7%, 95% CI 13.3–16.2) and 323/2238 (13.9%, 95% CI 12.5–15.3) HCWs, respectively. Between hospitals, the seroprevalence varied from 3.2% up to 30.0% (Additional file 1: Fig. S1).

Three months after enrolment, the prevalence of total antibodies increased by 1.4% (Fig. 1). In detail, seroconversion was observed for 21 (1.1%) of the 1881 seronegative HCWs, while seroreversion occurred in 3 (0.9%) of 339 seropositive HCWs with an available serum sample at the end of follow-up. Confirmatory testing for neutralising antibodies was not performed at follow-up. The number of seroconversions was too low to identify determinants associated with seroconversions.

### Determinants of seropositivity at baseline

The relative frequency of HCWs with positive and negative serology was described across levels of demographic, personal and occupational characteristics (Table 1).

Table 2 provides crude and adjusted ORs for the association between the presence of neutralising SARS-CoV-2 antibodies and determinants. Some work-related factors were among the strongest predictors of having antibodies: nurses (aOR 2.21, 95% CI 1.34–3.64) and administrative staff (aOR 1.87, 95% CI 1.02–3.43) had a higher seroprevalence than physicians. The emergency department (ED) (aOR 1.79, 95% CI 1.10–2.91) was the COVID-19-dedicated ward with the highest seroprevalence, whereas antibodies were less frequent in HCWs in the intensive, high, or medium care units (aOR 0.47, 95% CI 0.31–0.71). Chronic respiratory disease (aOR 0.52, 95% CI 0.28–0.96) and smoking (aOR 0.42, 95% CI 0.23–0.76) were associated with lower seroprevalence in our cohort, while diabetes mellitus was associated with higher seroprevalence (aOR 2.67, 95% CI 1.08–6.62). HCWs younger than 35 had increased odds (aOR 1.53, 95% CI 1.07–2.18) of being seropositive for SARS-CoV-2 compared to HCWs aged 36–49. Finally, owning a dog was associated with lower odds for seropositivity (aOR 0.65, 95% CI 0.46–0.92), while having a cat did not seem to play a role.

**Table 2:** Determinants of SARS-CoV-2 neutralising antibodies at baseline

Characteristic		Seroprevalence	Univariable analysis	Multivariable analysis^¥^
		(%)	Crude OR (95% CI)	Adjusted OR (95% CI)
Gender	Female	14.2	1	1
	Male	12.6	0.87 (0.63–1.18)	1.08 (0.77–1.52)
Age category	≤ 35	15.2	**1.45 (1.08–1.97)**	**1.53 (1.07–2.18)**
	36–49	11.0	1	1
	≥ 50	15.3	**1.47 (1.09–1.98)**	1.38 (0.96–1.99)
Hospital staff role	Physician	9.9	1	1
	Nurse	17.9	**1.98 (1.30–3.11)**	**2.21 (1.34–3.64)**
	Administrative	14.2	1.50 (0.96–2.43)	**1.87 (1.02–3.43)**
	Other	11.4	1.17 (0.76–1.85)	1.32 (0.79–2.20)
Direct contact with patients*	No patient contact	11.8	1	1
	With non-COVID patients	12.5	1.07 (0.75–1.54)	1.23 (0.78–1.92)
	With COVID patient	15.5	**1.38 (1.03–1.85)**	1.15 (0.73–1.79)
COVID-19 dedicated ward	Intensive to medium care	9.5	**0.61 (0.41–0.88)**	**0.47 (0.31–0.71)**
	Emergency	20.0	**1.61 (1.03–2.42)**	**1.79 (1.10–2.91)**
	Infectious disease	16.1	1.21 (0.75–1.87)	1.45 (0.85–2.48)
	Pulmonology	14.3	1.04 (0.68–1.54)	0.75 (0.47–1.21)
Smoker	No	14.9	1	1
	Former	13.2	0.86 (0.66–1.12)	0.83 (0.62–1.12)
	Current	7.7	**0.47 (0.26–0.80)**	**0.42 (0.23–0.76)**
Chronic respiratory disease		7.2	**0.46 (0.24–0.81)**	**0.52 (0.28–0.96)**
Diabetes Mellitus		21.9	1.75 (0.70–3.88)	**2.67 (1.08–6.62)**
Cardiovascular disease		16.8	1.29 (0.84–1.90)	1.98 (0.97–4.02)
Immune disorder		13.7	0.99 (0.40–2.07)	1.69 (0.66–4.32)
Use of NSAID		1.5	0.52 (0.18–1.18)	0.50 (0.19–1.34)
Use of antihypertensive medications		13.8	0.99 (0.59–1.58)	0.49 (0.21–1.13)
Use of immunosuppressants		5.3	0.34 (0.08–0.93)	0.31 (0.08–1.15)
Influenza vaccine season 2019/20		11.5	**0.68 (0.54–0.87)**	0.78 (0.59–1.01)
Travel to other European country*		15.3	1.17 (0.90–1.51)	1.28 (0.97–1.69)
Number of household members^#^	1	14.7	1	1
	2	12.6	0.84 (0.57–1.26)	0.75 (0.49–1.15)
	3	12.8	0.85 (0.54–1.36)	0.93 (0.56–1.56)
	4	15.1	1.03 (0.70–1.56)	1.15 (0.71–1.85)
	≥ 5	15.4	1.06 (0.66–1-69)	1.27 (0.74–2.16)
Children ≤ 11 yrs in the household	No	14.5	1	1
	Yes	13.9	0.82 (0.63–1.07)	0.75 (0.52–1.09)
Dog owner	No	14.4	1	1
	Yes	11.6	0.78 (0.56–1.06)	**0.65 (0.46–0.92)**
Cat owner	No	14.7	1	1
	Cat indoor only	11.5	0.75 (0.53–1.04)	0.81 (0.57–1.15)
	Cat in- and outdoor	10.1	0.65 (0.35–1.13)	0.75 (0.40–1.38)
Province of residence	North Brabant	19.5	1	1
	North Holland	11.7	**0.54 (0.30–0.91)**	0.49 (0.21–1.14)
	South Holland	10.3	**0.47 (0.34–0.65)**	**0.44 (0.26–0.74)**
	Gelderland	8.5	**0.38 (0.25–0.57)**	**0.36 (0.19–0.68)**
	Limburg	17.0	0.84 (0.56–1.25)	0.84 (0.40–1.76)
	Groningen	3.2	**0.14 (0.05–0.30)**	**0.13 (0.04–0.40)**

Bold font indicates statistical significance

*NSAID* non-steroidal anti-inflammatory drugs, *OR* odds ratio, *CI* confidence interval

*Since January 1, 2020. ^#^Including study participant. ^¥^Mixed effect logistic regression model with random intercept per hospital

The sensitivity analysis performed with seropositivity based on total SARS-CoV-2 antibodies as the outcome variable yielded similar results (Additional file 1: Table S1).

### Self-reported symptoms associated with seropositivity at baseline

Since January 2020 and before enrolment, 720 (30.9%) HCWs had been tested for SARS-CoV-2 infection, 668 (56.1%) of 1190 symptomatic HCWs and 52 (4.6%) of 1138 asymptomatic HCWs. The proportion of symptomatic HCWs with a positive PCR test was 65.8% among seropositives and 3.1% among seronegative subjects (Table 1). The main reason for having been tested was the presence of symptoms suggestive of SARS-CoV-2 infection (92.8%, n = 668). Other reasons for having been tested included: contact with a positive colleague (n = 61, 8.5%), contact with a positive patient (n = 61, 8.5%), contact with a positive person in the community (n = 23, 3.2%), scientific research (n = 26, 3.6%), and other reasons (n = 29, 4.0%).

In total, 1190 (51.1%) HCWs reported having experienced at least one COVID-19-related symptom since the beginning of January 2020, with cough (30.2%), general malaise/fatigue (27.3%) and runny nose (25.7%) being the most common. General malaise/fatigue (61.3%) and fever (53.6%) were the most common symptoms among seropositive HCWs. Forty-nine (15.2%) of the 323 seropositive HCWs reported no previous symptoms. Observed differences in the proportion of asymptomatic SARS-CoV-2 infections between age groups were not statistically significant (≤ 35 years: 14.8%; 36–49 years: 20.5%; ≥ 50 years: 12.0%, *p* = 0.245).

Only four of the 323 seropositive HCWs were hospitalised because of the reported symptoms, leading to a hospitalisation rate of 1.2% among seropositive HCWs. At baseline, the median time since the onset of self-reported symptoms was 100 days (IQR 79–116). The duration of self-reported symptoms was longer for seropositive than for seronegative HCWs (median, IQR: 2 weeks, 1–3 vs. 1 week, 0–3; *p* < 0.001).

Most self-reported symptoms were associated with a higher baseline seroprevalence in the univariable analysis, except for having a runny nose or a sore throat (Table 3). In the multivariable analysis, however, only altered or decreased smell (aOR 6.33, 95% CI 3.66–11.07), altered or decreased taste (aOR 2.25, 95% CI 1.29–3.86), fever (aOR 2.48, 95% CI 1.72–3.59), fatigue (aOR 1.55, 95% CI 1.04–2.31) and muscle aches (aOR 2.00, 95% CI 1.3–3.01) were independently associated with the development of neutralising antibodies, while sore throat (aOR 0.41, 95% CI 0.28–0.58) and chills (aOR 0.55, 95% CI 0.35–0.84) were negatively associated (Table 3).

**Table 3:**
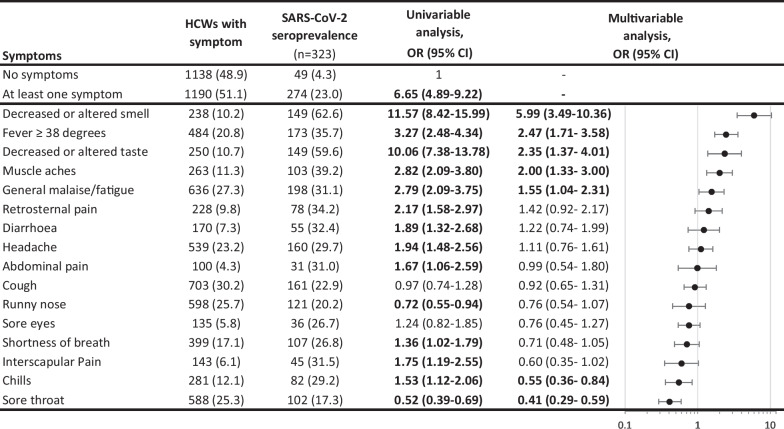
Prevalence of neutralising SARS-CoV-2 antibodies in HCWs and association measures per self-reported symptom

*HCWs* healthcare workers, *OR* odds ratio, *CI* confidence interval. Bold font indicates statistical significance.

## Discussion

In this multicentre seroepidemiology study in HCWs, the SARS-CoV-2 seroprevalence was 14% at the end of the first wave of the COVID-19 pandemic. The observed seroprevalence in HCWs was higher than that reported for the general population [13] but reflected the same regional differences [16, 28]. We detected a substantially higher SARS-CoV-2 seroprevalence among nurses, administrative and emergency department staff, young HCWs, and those with diabetes mellitus. On the other hand, lower seroprevalence was found in employees working in intensive, high or medium care and those with self-reported lung disease, smokers, and dog owners. Notably, 15% of the seropositive participants had an asymptomatic course of infection.

### SARS-CoV-2 seroprevalence

In our study, the estimated seroprevalence in HCWs at enrolment was three times higher than that measured in the general population [13] and healthy donors [15] around the same period. Even though the actual prevalence might have been lower due to potential selective participation in the study, our result confirms that HCWs were exposed to a high risk of infection in the first phase of the COVID-19 pandemic. The differences in seroprevalence between hospitals reflect regional differences in the incidence of SARS-CoV-2 infections in the Netherlands in the first months of the pandemic; this geographical correlation might be caused by a higher exposure in the community and a higher number of infectious patients to care for in the workplace. Our seroprevalence rates in HCWs per province align with the results of two studies performed among Dutch HCWs in the Amsterdam area [18] and Limburg [17]. Additionally, we provide data on HCWs working in other provinces with different SARS-CoV-2 incidences.

We observed that almost all participants with binding antibodies at baseline also showed neutralising antibodies, confirming the findings from earlier studies [29, 30]. Conversely, a German population-based study reported detectable neutralising antibodies in only one-third of the participants with a positive immunoassay result [31]. This inconsistency could be due to the use of different assays or a different timing from infection onset to the moment of the serological testing between the studies.

SARS-CoV-2 seroprevalence in HCWs showed a limited increase during the three-month follow-up, suggesting that the viral circulation among Dutch HCWs was minimal in the summer. Only 1% of the tested participants seroconverted. The SARS-CoV-2 seroreversion rate after follow-up was very low, suggesting that antibody titres remain relatively stable for several months, even after mild infections, as demonstrated in other studies [29, 32].

### Determinants associated with seropositivity at baseline

No difference in seroprevalence was observed between men and women; this was in line with the conclusion of a living systematic review that found no consistent association between risk for SARS-CoV-2 infection in HCWs and sex [19].

We identified occupational risk and protective factors that could inform risk assessment for staff and be the target of hospital infection control policies. HCWs in the ED had the highest seroprevalence, and those working in intensive, high, or medium care had the lowest. Prior studies [33, 34] also described a higher risk among the ED staff, which might be explained by the frequent exposure, sometimes without adequate PPE, to patients with asymptomatic or undiagnosed SARS-CoV-2 infection, and by the overcrowding of these wards during the pandemic. Especially at the beginning of 2020, the definition of a suspected COVID-19 case was very stringent and tied to the epidemiological criterion (e.g., contact with a known infected case or travel history to high-risk areas) [35]; the PPE supply was limited and prioritised for the workers exposed to confirmed or suspected COVID-19 patients. As a result, ED employees might have had unprotected exposure to patients unsuspected of COVID-19.

Lower odds of infection among HCWs in intensive, high, or medium care than among HCWs in other hospital wards were previously described in other countries [33, 36–38]. This finding suggests that in certain working conditions (e.g., low staff-to-patient ratio, staff aware of the risk of infection, availability and correct use of adequate PPE), viral transmission can be prevented; this would apply even when aerosol-generating procedures are performed, as usually happens in intensive care units (ICU). An additional explanation might be the different phase of the disease patients are in when being admitted to ICU (i.e., later in the COVID-19 course with lower viral loads) compared to the average patient visiting the emergency department.

In line with prior studies, we found that nurses were more likely to be seropositive for SARS-CoV-2 antibodies than physicians [4, 33, 39–41]. Assuming that all healthcare roles had equitable access to PPE across the Dutch hospitals, the higher risk for nurses might be attributed to the frequency, intensity and duration of patient contacts.

The observation that administrative employees had a higher seroprevalence than physicians points out that HCWs can acquire the infection through ways other than direct patient care, such as exposure to co-workers, household members, or persons in the community [21, 23, 42–44].

One of the questions that produced the most conflicting results is whether caring for known COVID-19 patients increased the risk of acquiring the infection compared to not working in COVID-19-dedicated units [11, 12, 19, 39, 45]. In our study, univariable analysis showed that HCWs having contact with known infected patients had higher odds of infection than those not having contact with patients. After taking into account differences in baseline risk between hospitals and adjusting for confounders and community risk (province), we found no increased risk in HCWs exposed to COVID-19 patients. This finding, together with the low risk described in intensive, high or medium care employees, points to the fact that HCWs aware of the risk of infection and applying the correct use of PPE were able to reduce the risk of becoming infected while caring for COVID-19 patients.

Previous studies reported that young people have the highest rate of COVID-19 infections, probably due to age-dependent social behaviours [16, 28]. Our data present a similar trend to that of the Dutch general population: the highest prevalence of antibodies in the younger group, followed by that in the older group, and finally, the mid-aged group that showed the lowest rate, even after adjusting for working-related covariates (Table 2). Other seroprevalence studies among HCWs also found that younger staff were more likely to be seropositive [21, 34, 46]. We hypothesise that these differences might be influenced by different social behaviours of HCWs outside the hospital, highlighting the contribution of community acquisition of the virus among hospital staff. A recent Dutch study attributed the higher seroprevalence in young HCWs to increased participation in Carnival festivities [17], celebrated in the southern part of the country at the end of February 2020.

Another possible explanation for the lowest presence of antibodies in the mid-age group could be the cross-protective immunity to SARS-CoV-2 offered by the presence of young children in the household, as suggested by some studies [47–49]. Two studies showed that the number of young children in the household was inversely associated with the risk of infection in HCWs [48, 50]. We did not find significant differences in seroprevalence between HCWs living with and without young children (≤ 11 years), but the model estimate pointed towards lower odds of seropositivity in those living with young children. Prior studies suggested that big households had a higher risk of COVID-19 infection [51, 52]. In our study, the odds of having antibodies showed an increasing trend for households with ≥ 4 members, but the association did not reach statistical significance.

Our data suggest that recent influenza vaccination may be associated with a reduced risk of SARS-CoV-2 infection, even though the association was not statistically significant after confounder adjustment. Our result is in line with the growing body of evidence hinting at the non-specific protective effects of influenza vaccination against COVID-19-related outcomes [53–57]. Induced trained immunity has been suggested to have a role in such a protective effect, but the underlying mechanism is not fully understood, and the duration of protection is unclear [58, 59]. A more prudent behaviour aimed at reducing the risk of being infected with SARS-CoV-2 (e.g., by wearing PPE properly and respecting social distancing) could characterise the HCWs who sought a seasonal flu shot compared to the unvaccinated. This behaviour, defined as the ‘healthy-user effect’, could have contributed to the lower risk in this group.

Similarly, this preventive attitude could partially explain the lower seroprevalence in employees with self-reported respiratory disease. Concerned about a higher risk of severe COVID because of the chronic disease, they might have generally been more cautious during the pandemic. In line with the conclusion of a recent systematic review claiming that people with asthma bear a reduced risk of being infected with SARS-CoV-2 [60], we found that the odds of being seropositive were 50% lower for HCWs with a respiratory condition, being mainly asthma (88%) in our cohort. Previous studies suggested that the prevalence of chronic respiratory disease in patients hospitalised with COVID-19 was lower than in the general population [61] and that pre-existing respiratory conditions are not associated with an increased risk of severe COVID as initially expected [62]. The reduced susceptibility to SARS-CoV-2 infection in asthma patients may be attributed to the altered expression of the angiotensin-converting enzyme2 (ACE2) receptor in their lower airway [63]. It has also been suggested that treatments used by patients with chronic respiratory diseases could decrease the risk of infection, but studies yielded conflicting results, and evidence is lacking [64–66].

We detected a significantly higher SARS-CoV-2 seropositivity in participants with diabetes mellitus. This finding is in line with previous studies pointing toward an increased SARS-CoV-2 susceptibility for subjects with this chronic condition, assessed before the launch of vaccination programs [67]. We observed a lower seroprevalence among active smokers, confirming the findings of multiple other studies summarised in a systematic review [68], including studies in HCWs [50, 69, 70]. However, it seems counterintuitive to presume a protective role of smoking from these studies, considering the expected greater risk of worse outcomes among smokers with COVID-19 [71]. The evidence of an increased risk of severe course of disease among infected smokers is inconclusive, and the pathogenetic mechanism underlying the relationship between smoking and COVID-19 is still unclear [72, 73]. Most studies on the topic, including our study, adopted observational designs, which may be prone to selection bias, making it uncertain whether the association is causal or just the result of a confounder effect [72, 74]. Behavioural factors may also explain the preventive effect of active smoking in HCWs, as spending breaks outdoor smoking might have helped to avoid high-risk exposure to colleagues in common areas. In addition to reduced susceptibility to SARS-CoV-2 infection, we hypothesise that smokers might have an impaired ability to produce antibodies. This hypothesis follows the results of a Spanish seroprevalence study that looked at both COVID-19 diagnosis and the presence of antibodies; the authors found that the difference between the proportion of seropositive subjects and diagnoses was lower for smokers (12.4%) than for non-smokers (16.3%), possibly because some subjects among the smokers did not develop antibodies [75].

Finally, this is the first study highlighting a reduced risk of COVID-19 in dog owners. While there are studies describing a higher likelihood of SARS-CoV-2 infection in pets living in households with infected humans [76, 77], we did not identify any studies exploring the effect of having a pet on COVID-19 risk in humans. Dogs can be infected by humans, but (unlike cats) there is no evidence that they contribute to further transmission of the virus [77]. We think that dog owners might have certain behaviours or lifestyles that expose them to a lower risk of infection (e.g., walking the dog vs. gathering with friends), rather than the dog itself being a protective factor. However, we can only speculate about the underlying mechanisms of this association, which might also be the effect of residual confounding.

### Symptoms associated with seropositivity

The majority of HCWs with SARS-CoV-2 antibodies suffered from mild disease. The absence of symptoms for 15% of the employees who had developed antibodies was remarkable. The rate of asymptomatic infections found in our study is comparable with the rate (16%) described by Bouwman et al. [17] in a cohort of HCWs of a teaching hospital in the Netherlands after the first wave and to those reported in other countries [43]. A few other studies found a proportion of asymptomatic infections among HCWs of about 30% [45, 78, 79], probably because we investigated a more comprehensive range of symptoms. It is also possible that some asymptomatic infections occurring in early March 2020 resulted in a negative serology test at the time of study enrolment. Nevertheless, the proportion of asymptomatic infections among all confirmed cases differs significantly in the available literature and can be influenced by many factors, as highlighted by a recent article summarising the results of 14 systematic reviews on the topic [80].

Only 16.8% of the HCWs who were tested since January 1, 2020 because of self-reported COVID-19 suspected symptoms reported a SARS-CoV-2 positive RT-PCR test result, indicating the limited specificity of symptoms.

In our study, the first 200 respondents were enrolled in each hospital. Voluntary participation might have led to selection bias, i.e., HCWs with previously confirmed or suspected COVID-19 might have participated more likely, and those with severe disease less likely. This might explain the observed low hospitalisation rate in our study compared to the HCWs’ hospitalisation and mortality rates of 15.1% and 1.5%, respectively, reported in a systematic review [81].

For the period since January 1, 2020, employees with SARS-CoV-2 antibodies reported a higher number of and a longer duration of symptoms than employees for whom no antibodies could be detected.

We confirmed altered or decreased smell and taste as specific symptoms of infection with the Wuhan wild-type strain of SARS-CoV-2 [3, 45, 79, 82]. In addition, fever, muscle aches, and fatigue were independent predictors of SARS-CoV-2 antibodies in our cohort of non-vaccinated HCWs with mild COVID-19 infection, while sore throat and chills seemed predictive of other conditions, as described by other studies [45, 79, 82]. However, as the clinical picture has appeared to evolve with new SARS-CoV-2 variants [83, 84], the context and the predominant variant should be taken into account when considering a set of symptoms for the prediction of SARS-CoV-2 infection.

### Strengths and limitations

This study presents some limitations. First, the cross-sectional design makes it challenging to claim causality between exposures and seropositivity, and the retrospective and self-reported data collection might have introduced information and recall bias. Second, the voluntary participation in the study might have led to selection bias, leading to an overestimation of the seroprevalence and an overrepresentation of mild cases. Third, seroprevalence was used as a proxy for infection, and, therefore, some infections might have been missed, as not all infected patients develop antibodies against SARS-CoV-2 [85]. Finally, the availability and use of PPE, which undoubtedly played a role in the risk of contracting the virus after exposure, were not investigated.

The main strength of our study is that it represents the most extensive data collection of seroprevalence in HCWs in the Netherlands, with the participation of 13 hospitals across the country from areas with different disease incidences during the first wave. Second, plaque reduction neutralisation assays were applied. Neutralisation assays are the current gold standard for SARS-CoV-2 serology, as the presence of antibodies that can neutralise the virus is highly specific for prior infection and predictive of protective immunity [10, 31, 85, 86]. However, they are laborious and, therefore, less often applied in serosurveys than binding assays, making the determinants for the presence of SARS-CoV-2 neutralising antibodies relatively unexplored [18]. Additionally, our study reports detailed data on clinical presentation and seroprevalence and includes a wide range of potential community sources of exposure in addition to occupational factors.

### Recommendations

For future SARS-CoV-2 waves or pandemics, infection control in the emergency department should be reinforced, and the working conditions of nurses should be closely monitored. Additionally, training on correct PPE use should be enhanced and universal masking considered. Young HCWs shall be warned of their potential role in introducing the virus into healthcare facilities. Given the possible protective effect of influenza vaccination on infection acquisition, policymakers might consider implementing strategies to improve its uptake and promote its benefits for preventing and controlling COVID-19. Further research is nevertheless warranted to confirm the association and shed light on the underlying mechanism. Similarly, it would be interesting to better understand the relationship between COVID-19 and smoking and the possible cross-immunity related to children to evaluate and develop possible preventive strategies.

## Conclusions

In June-July 2020, at the end of the first wave of the SARS-CoV-2 pandemic, a SARS-COV-2 seroprevalence of 15% was observed in HCWs of 13 Dutch hospitals. During the three-month follow-up, only 1% of the tested participants seroconverted. Higher proportions of HCWs with SARS-CoV-2 antibodies were detected in the ED and among nurses, administrative and young staff, and those with diabetes mellitus, while a lower seroprevalence was found in HCWs in intensive, high, or medium care, and those with self-reported lung disease, smokers, and dog owners. The results of this study can be used to prioritise infection control and other preventive measures to protect HCWs at the highest risk, even when SARS-CoV-2 vaccines are available. A history of altered or decreased smell or taste, fever, muscle aches and fatigue were independently associated with the presence of SARS-CoV-2 antibodies in unvaccinated HCWs, while sore throat and chills were predictive of other conditions.

### Supplementary Information

**Additional file 1 Figure S1.** Seroprevalence per hospital. **Table S1.** Sensitivity analysis: determinants of total SARS-CoV-2 antibodies.

## Data Availability

The datasets generated and analysed during the current study are available from the corresponding author or the principal investigator on reasonable request.

## Abbreviations

ACE2: Angiotensin-converting enzyme 2
aOR: Adjusted odds ratios
BCG: Bacillus Calmette–Guérin
CI: Confidence interval
COCON: Control of COVID-19 in hospitals
COVID-19: Coronavirus disease 2019
ED: Emergency department
HCW: Healthcare workers
IQR: Interquartile range
NSAID: Non-steroidal anti-inflammatory drugs
RT-PCR: Reverse transcription-polymerase chain reaction
PPE: Personal protective equipment
RIVM: National Institute for Public Health and the Environment
SARS-CoV-2: Severe acute respiratory syndrome coronavirus 2
