# Supplementary material for: Seroprevalence of SARS-CoV-2 antibodies among healthcare workers in Dutch hospitals after the 2020 first wave: a multicentre cross-sectional study with prospective follow-up
Source: Antimicrob Resist Infect Control. 2023 Nov 29;12:137. doi: 10.1186/s13756-023-01324-x (PMC10688070; doi:10.1186/s13756-023-01324-x)
Supplement: Supplementary file 1 — Additional file 1 Figure S1. Seroprevalence per hospital. Table S1. Sensitivity analysis: determinants of total SARS-CoV-2 antibodies. [file 13756_2023_1324_MOESM1_ESM.docx]

Supplementary file to:

**Seroprevalence of SARS-CoV-2 antibodies among healthcare workers in Dutch hospitals after the 2020 first wave: a multicentre cross-sectional study with prospective follow-up**

Claudia Recanatini, Corine H. GeurtsvanKessel, Suzan D. Pas, Els M. Broens, Martje Maas, Rosa van Mansfeld, Anne J.G. Mutsaers-van Oudheusden, Miranda van Rijen, Emile F. Schippers, Arjan Stegeman, Adriana Tami, Karin Ellen Veldkamp, Hannah Visser, Andreas Voss, Marjolijn C.A. Wegdam-Blans, Heiman F.L. Wertheim, Peter C. Wever, Marion P.G. Koopmans, Jan A.J.W. Kluytmans, Marjolein F.Q. Kluytmans-van den Bergh, and COCON Study Group

Figure S1. Seroprevalence per hospital based on anti-SARS-CoV-2 neutralising antibodies

Table S1. Sensitivity analysis: distribution of subjects’ characteristics by serostatus, total antibodies (n and %), and risk factor analysis for having SARS-CoV-2 total antibodies at baseline. Bold font indicates statistical significance. *Since January 1, 2020. ^#^Including study participant. ^¥^Mixed effect logistic regression model with random intercept per hospital. BCG: Bacillus Calmette-Guérin. NSAID: non-steroidal anti-inflammatory drugs. *OR*: odds ratio. *CI*: confidence interval

| **Characteristic** |  | **Seronegative  (n=1985)** | **Seropositive  (n=343)** | **Seroprevalence (%)** | **Crude OR  (95% CI)** | **Adjusted OR  (95% CI)^¥^** |
| --- | --- | --- | --- | --- | --- | --- |
| Gender | Female | 1614 (81.3) | 284 (82.8) | 13.7 | 1 | 1 |
|  | Male | 371 (18.7) | 59 (17.2) | 15.0 | 0.90 (0.66-1.21) | 1.11 (0.80-1.55) |
| Age category | ≤ 35 | 634 (31.9) | 123 (35.9) | 16.2 | **1.47 (1.10-1.98)** | **1.52 (1.08-2.15)** |
|  | 36-49 | 668 (33.7) | 88 (25.7) | 11.6 | 1 | 1 |
|  | ≥ 50 | 683 (34.4) | 132 (38.5) | 16.2 | **1.47 (1.10-1.97)** | 1.41 (0.98-2.01) |
| Hospital staff role | Physician | 250 (12.6) | 32 (9.3) | 11.3 | 1 | 1 |
|  | Nurse | 592 (28.9) | 139 (40.5) | 19.0 | **1.83 (1.23-2.81)** | **2.07 (1.29-3.33)** |
|  | Administrative staff | 426 (21.5) | 73 (21.3) | 14.6 | 1.34 (0.87-2.11) | 1.69 (0.95-3.02) |
|  | Other supporting staff | 717 (36.1) | 99 (28.9) | 12.1 | 1.08 (0.71-1.67) | 1.22 (0.75-1.98) |
| Direct contact  with patients* | No patient contact | 537 (27.1) | 75 (21.9) | 12.3 | 1 | 1 |
|  | With non-COVID patients | 435 (21.9) | 69 (20.1) | 13.7 | 1.14 (0.80-1.61) | 1.27 (0.82-1.96) |
|  | With COVID patients | 1013 (51.0) | 199 (58.0) | 16.4 | **1.41 (1.06-1.88)** | 1.17 (0.76-1.80) |
| COVID-19  dedicated ward | Intensive to medium care | 313 (15.8) | 34 (9.9) | 9.8 | **0.59 (0.40-0.84)** | **0.46 (0.30-0.69)** |
|  | Emergency | 115 (5.8) | 30 (8.7) | 20.7 | **1.56 (1.01-2.34)** | **1.80 (1.11-2.90)** |
|  | Infectious disease | 125 (6.3) | 24 (7.0) | 16.1 | 1.12 (0.70-1.73) | 1.22 (0.72-2.07) |
|  | Pulmonology | 171 (8.6) | 32 (9.3) | 15.8 | 1.09 (0.72-1.60) | 0.82 (0.52-1.29) |
| Smoker | No | 1256 (63.3) | 236 (68.8) | 15.8 | 1 | 1 |
|  | Former | 561 (28.3) | 92 (26.8) | 14.1 | 0.87 (0.67-1.13) | 0.85 (0.64-1.13) |
|  | Current | 168 (8.5) | 15 (4.4) | 8.2 | **0.48 (0.26-0.79)** | **0.43 (0.24-0.76)** |
| Chronic respiratory disease | No | 1833 (92.3) | 329 (95.9) | 15.2 | 1 | 1 |
|  | Yes | 152 (7.7) | 14 (4.1) | 8.4 | **0.51 (0.28-0.87)** | 0.58 (0.32-1.04) |
| Diabetes mellitus | No | 1960 (98.7) | 336 (98.0) | 14.6 | 1 | 1 |
|  | Yes | 25 (1.3) | 7 (2.0) | 21.9 | 1.63 (0.65-3.61) | 2.44 (0.99-6.03) |
| Cardiovascular  disease | No | 1833 (92.3) | 311 (90.7) | 14.5 | 1 | 1 |
|  | Yes | 152 (7.7) | 32 (9.3) | 17.4 | 1.24 (0.82-1.83) | 2.11 (1.06-4.22) |
| Immune disorder | No | 1941 (97.8) | 336 (98.0) | 14.8 | 1 | 1 |
|  | Yes | 44 (2.2) | 7 (2.0) | 13.7 | 0.92 (0.38-1.93) | 1.45 (0.57-3.68) |
| Use of NSAID | No | 1926 (97.0) | 338 (98.5) | 14.9 | 1 | 1 |
|  | Yes | 59 (3.0) | 5 (1.5) | 7.8 | 0.48 (0.17-1.10) | 0.47 (0.18-1.24) |
| Use of antihypertensive medications | No | 1860 (93.7) | 323 (94.2) | 14.8 | 1 | 1 |
|  | Yes | 125 (6.3) | 20 (5.8) | 13.8 | 0.92 (0.55-1.47) | **0.43 (0.19-0.99)** |
| Use of immunosuppressants | No | 1932 (97.3) | 339 (98.8) | 14.9 | 1 | 1 |
|  | Yes | 53 (2.7) | 4 (1.2) | 7.0 | 0.43 (0.13-1.06) | 0.43 (0.14-1.38) |
| BCG vaccine  received between  1940 and 2020 | No | 1490 (85.7) | 249 (14.3) | 14.3 | - | - |
|  | Yes | 219 (87.3) | 32 (12.7) | 12.7 | - | - |
|  | Unknown | 276 (81.7) | 62 (18.3) | 18.3 | - | - |
| Influenza vaccine  season 2019/20 | No | 1054 (53.1) | 210 (61.2) | 16.6 | 1 | 1 |
|  | Yes | 931 (46.9) | 133 (38.8) | 12.5 | **0.72 (0.57-0.91)** | 0.79 (0.61-1.02) |
| Travel to other European country* | No | 1457 (85.8) | 242 (14.2) | 14.2 | 1 | 1 |
|  | Yes | 528 (83.9) | 101 (16.1) | 16.1 | 1.15 (0.89-1.48) | 1.23 (0.94-1.62) |
| Number of  household members^#^ | 1 | 225 (11.3) | 41 (12.0) | 15.4 | 1 | 1 |
|  | 2 | 679 (34.2) | 108 (31.5) | 13.7 | 0.87 (0.60-1.30) | 0.78 (0.51-1.18) |
|  | 3 | 306 (15.4) | 46 (13.4) | 13.1 | 0.82 (0.52-1.30) | 0.88 (0.53-1.46) |
|  | 4 | 532 (26.8) | 98 (28.6) | 15.6 | 1.01 (0.68-1.52) | 1.11 (0.70-1.76) |
|  | ≥ 5 | 243 (12.2) | 50 (14.6) | 17.1 | 1.13 (0.72-1.78) | 1.33 (0.79-2.23) |
| Children ≤11 years  in the household | No | 1405 (70.8) | 255 (74.3) | 15.4 | 1 | 1 |
|  | Yes | 580 (29.2) | 88 (25.7) | 13.2 | 0.84 (0.64-1.08) | 0.79 (0.55-1.13) |
| Dog owner | No | 1589 (80.1) | 289 (84.3) | 15.4 | 1 | 1 |
|  | Yes | 396 (19.9) | 54 (15.7) | 12.0 | 0.75 (0.54-1.02) | **0.64 (0.46-0.90)** |
| Cat owner | No | 1524 (76.8) | 282 (82.2) | 15.6 | 1 | 1 |
|  | Cat indoor only | 115 (5.8) | 14 (4.1) | 10.9 | 0.66 (0.36-1.12) | 0.79 (0.56-1.11) |
|  | Cat in- and outdoor | 346 (17.4) | 47 (13.7) | 12.0 | 0.73 (0.52-1.01) | 0.75 (0.41-1.36) |
| Province of residence | North Brabant | 738 (37.2) | 189 (55.1) | 20.4 | 1 | 1 |
|  | North Holland | 120 (6.0) | 17 (5.0) | 12.4 | **0.55 (0.31-0.92)** | 0.51 (0.24-1.12) |
|  | South Holland | 495 (24.9) | 59 (17.2) | 10.6 | **0.47 (0.34-0.63)** | **0.44 (0.27-0.71)** |
|  | Gelderland | 315 (15.9) | 38 (11.1) | 10.8 | **0.47 (0.32-0.68)** | **0.45 (0.25-0.80)** |
|  | Limburg | 166 (8.4) | 34 (9.9) | 17.0 | 0.80 (0.53-1.18) | 0.79 (0.40-1.58) |
|  | Groningen | 151 (7.6) | 6 (1.7) | 3.8 | **0.16 (0.06-0.33)** | **0.15 (0.05-0.42)** |
